# Supplementary material for: Health care utilization at end of life among patients with lung or pancreatic cancer. Comparison between two Swedish cohorts
Source: PLoS One. 2021 Jul 16;16(7):e0254673. doi: 10.1371/journal.pone.0254673 (PMC8284833; doi:10.1371/journal.pone.0254673)
Supplement: S1 Fig — A Diagram displaying the inclusion process and reasons for exclusion, as well as the total cohort included. (DOCX) [file pone.0254673.s001.docx]

Figure 1 supplementary material, description of the selection process for the included patients
