# Supplementary material for: Health care utilization at end of life among patients with lung or pancreatic cancer. Comparison between two Swedish cohorts
Source: PLoS One. 2021 Jul 16;16(7):e0254673. doi: 10.1371/journal.pone.0254673 (PMC8284833; doi:10.1371/journal.pone.0254673)
Supplement: Attachment — Submitted filename: Response to reviewers.docx [file pone.0254673.s002.docx]

PONE-D-21-08049
Health care utilization at end of life among patients with lung or pancreatic cancer. Comparison between two Swedish cohorts
PLOS ONE

Dear Dr. Ullgren,

Thank you for submitting your manuscript to PLOS ONE. After careful consideration, we feel that it has merit but does not fully meet PLOS ONE’s publication criteria as it currently stands. Therefore, we invite you to submit a revised version of the manuscript that addresses the points raised during the review process:

Specifically:

1-For readers who are not familiar with the Swedish health care system, please elaborate on the quality and accessibility of the study population to the specialized palliative care (SPC)  as in/out- patient. What would SPC at the home cover, which is different from in-patient palliative care services and resulting in decreased numbers of acute hospital beds?

Thank you for pointing this out. This was a strategic decision from health care officials, to decentralise cancer away from the hospitals to get closer to the patients. As a result, the number of hospital beds were reduced. SPC at home should include inpatient services, at hospice for instance. We have tried to clarify the introduction in the second paragraph, page 4, starting from line 66 throughout 75 marked in red.

2- Please be specific on the integration of EOL care and how the increased use of SACT, ED visits, ICU care, and death in other care settings endanger this integration?

Thank you, this is important and we have tried to clarify and elaborated on this in the discussion section, page 12, paragraph 4, starting on line 264 to 271- marked in red.

3- Please address comments by reviewers #1,2

We have addressed these, see below, under each reviewers comments.

We also took the liberty to (just after Table 1) add footnotes that was not visible in manuscript submitted, see marked in red at line 161-166, as well as clarification of a sentence in the abstract, that was not clear, page 3, first paragraph, line 41-43

1) Please ensure that your manuscript meets PLOS ONE's style requirements, including those for file naming.

Thank you! We have read those and re-named the files accordingly, and added in the end of the abstract, page 20, “Supporting information” on S1Fig. line 512-514.

2) Please provide additional details regarding participant consent. In the ethics statement in the Methods and online submission information, please ensure that you have specified (1) whether consent was informed and (2) what type you obtained (for instance, written or verbal, and if verbal, how it was documented and witnessed). If your study included minors, state whether you obtained consent from parents or guardians. If the need for consent was waived by the ethics committee, please include this information.

Thank you for noticing that we were not clear. We have no informed consents, since we only used register data on deceased patients.

If you are reporting a retrospective study of medical records or archived samples, please ensure that you have discussed whether all data were fully anonymized before you accessed them and/or whether the IRB or ethics committee waived the requirement for informed consent. If patients provided informed written consent to have data from their medical records used in research, please include this information.

The ethics committee does not require informed consents for using registry data, and all data was anonymized before analysis. We have in the manuscript, in methods, page 5, third paragraph, on line, 97-100 added a small section, explaining the linking procedure, and how we did to ensure autonomy. We have not accessed patients’ medical records- only data pulled from registries. To be more clear, we have changed a word in methods section, page 5, second paragraph, line 93- from database to registry (marked in red).

3)  We note that you have indicated that data from this study are available upon request. PLOS only allows data to be available upon request if there are legal or ethical restrictions on sharing data publicly. For information on unacceptable data access restrictions, please see <http://journals.plos.org/plosone/s/data-availability#loc-unacceptable-data-access-restrictions>.

We are sorry and apologize, for being unclear. We do believe that all relevant data are within the manuscript and supporting files. It isn’t allowed under Swedish Ethical law to share data, when it involves information on humans, however we have provided the contact information to the Ethics board, since all requests for data must go through them.

In your revised cover letter, please address the following prompts:

a) If there are ethical or legal restrictions on sharing a de-identified data set, please explain them in detail (e.g., data contain potentially identifying or sensitive patient information) and who has imposed them (e.g., an ethics committee). Please also provide contact information for a data access committee, ethics committee, or other institutional body to which data requests may be sent.

I will address this in the cover letter, and as mentioned above, no data may be shared according to Swedish ethical regulations. Any request must go through the Ethics board. They may be contacted here;

[registrator@etikprovning.se](mailto:registrator@etikprovning.se)

+46 (0)10-475 08 00

b) If there are no restrictions, please upload the minimal anonymized data set necessary to replicate your study findings as either Supporting Information files or to a stable, public repository and provide us with the relevant URLs, DOIs, or accession numbers. Please see <http://www.bmj.com/content/340/bmj.c181.long> for guidelines on how to de-identify and prepare clinical data for publication. For a list of acceptable repositories, please see <http://journals.plos.org/plosone/s/data-availability#loc-recommended-repositories>.

We will update your Data Availability statement on your behalf to reflect the information you provide.

Thank you for this – let us know if anything is still unclear. Please see our attempt of revising the data-availability statement accordingly.

4) We note that you have included the phrase “data not shown” in your manuscript. Unfortunately, this does not meet our data sharing requirements. PLOS does not permit references to inaccessible data. We require that authors provide all relevant data within the paper, Supporting Information files, or in an acceptable, public repository. Please add a citation to support this phrase or upload the data that corresponds with these findings to a stable repository (such as Figshare or Dryad) and provide and URLs, DOIs, or accession numbers that may be used to access these data. Or, if the data are not a core part of the research being presented in your study, we ask that you remove the phrase that refers to these data.

This is not an essential part of our study and we removed this sentence, page 7, last paragraph, line 153-154..

[Note: HTML markup is below. Please do not edit.]

Reviewers' comments:

Reviewer's Responses to Questions

**Comments to the Author**

1. Is the manuscript technically sound, and do the data support the conclusions?

The manuscript must describe a technically sound piece of scientific research with data that supports the conclusions. Experiments must have been conducted rigorously, with appropriate controls, replication, and sample sizes. The conclusions must be drawn appropriately based on the data presented.

Reviewer #1: Yes

Reviewer #2: Yes

2. Has the statistical analysis been performed appropriately and rigorously?

Reviewer #1: Yes

Reviewer #2: Yes

3. Have the authors made all data underlying the findings in their manuscript fully available?

The [PLOS Data policy](http://www.plosone.org/static/policies.action#sharing) requires authors to make all data underlying the findings described in their manuscript fully available without restriction, with rare exception (please refer to the Data Availability Statement in the manuscript PDF file). The data should be provided as part of the manuscript or its supporting information, or deposited to a public repository. For example, in addition to summary statistics, the data points behind means, medians and variance measures should be available. If there are restrictions on publicly sharing data—e.g. participant privacy or use of data from a third party—those must be specified.

Reviewer #1: Yes

Reviewer #2: Yes

4. Is the manuscript presented in an intelligible fashion and written in standard English?

PLOS ONE does not copyedit accepted manuscripts, so the language in submitted articles must be clear, correct, and unambiguous. Any typographical or grammatical errors should be corrected at revision, so please note any specific errors here.

Reviewer #1: Yes

Reviewer #2: Yes

5. Review Comments to the Author

Please use the space provided to explain your answers to the questions above. You may also include additional comments for the author, including concerns about dual publication, research ethics, or publication ethics. (Please upload your review as an attachment if it exceeds 20,000 characters)

Reviewer #1: The study emphasizes the importance of integrating a palliative care approach, including conversations about care goals and preferred place of death. In addition, better psychosocial support and care coordination are important factors in avoiding acute care at EOL. Another interesting change was the fact that a higher percentage of patients died in non-acute settings. In terms of the organization of EOL care, the results show an increase in the use of SPC at home over time. Despite this, our findings raise questions about how well-integrated care is, as the use of SACTs, ED visits, ICU care and death in other care settings has increased. the study found that communication between acute and palliative health organizations is inadequate and has an impact on unplanned acute hospital admissions.
Research which I hope will be a stimulus for health care organizations and stakeholders to better organize and coordinate care for vulnerable patients

Thank you for this, and we agree.

Reviewer #2: An extremely clear and well written report.

I do wonder about the availability of data regarding provision of care and potential differences between 2010 and 2017: E.g. in the discussion section, page 11 starting in line 244: could authors comment regarding provision of care, mainly a) healthcare professionals palliative care training differences between 2010 and 2017 and b) the existing number of home care teams between 2010 and 2017 across the region and if these could have affected results in any way

Thank you for this valuable comment; we have tried to elaborate on this, page 13, first paragraph now in lines 273- 278, in the discussion section.

There are quite a few typos. This is by no means an exhaustive list.
Page 3 line 53 “and to be to able prepare and discuss the purpose and priorities at EOL”
Page 3 line 57 “but a set of measures is widely used”
Page 4 line 86 “Diagnostic data were retrieved”
Page 4 line 92 “patients with lung or pancreatic cancer who died in Stockholm”
And so on

Thank you for this, the manuscript has been carefully revised throughout for typos.

6. PLOS authors have the option to publish the peer review history of their article ([what does this mean?](https://journals.plos.org/plosone/s/editorial-and-peer-review-process#loc-peer-review-history)). If published, this will include your full peer review and any attached files.

We agree to publish peer review history.
If you choose “no”, your identity will remain anonymous but your review may still be made public.


**Do you want your identity to be public for this peer review?** For information about this choice, including consent withdrawal, please see our [Privacy Policy](https://www.plos.org/privacy-policy).

Reviewer #1: **Yes:** Cascioli Marta

Reviewer #2: No

[
